# First Steps in the Successful Fertilization of Rice and *Arabidopsis*: Pollen Longevity, Adhesion and Hydration

**DOI:** 10.3390/plants9080956

**Published:** 2020-07-29

**Authors:** Sunok Moon, Ki-Hong Jung

**Affiliations:** Graduate School of Biotechnology and Crop Biotech Institute, Kyung Hee University, Yongin 17104, Korea; moonsun@khu.ac.kr

**Keywords:** pollen, rice, dehiscence, pollen hydration, omics data

## Abstract

Understanding the behavior of pollen during pollination is important for food security in the future. The elucidation of pollen development and growth regulation largely relies on the study of the dicotyledonous model plant *Arabidopsis thaliana*. However, rice (*Oryza sativa*) pollen exhibits different characteristics to that of *Arabidopsis*. The latter undergoes programmed dehydration and withstands adverse environmental conditions, whereas rice pollen is sensitive to desiccation. Moreover, the short longevity of rice pollen significantly hampers hybrid seed production. Although the “omics” data for mature rice pollen have been accumulated, few genes that control pollination and pollen hydration have been identified. Therefore, to facilitate future studies, it is necessary to summarize the developmental processes involved in pollen production in rice and to consolidate the underlying mechanisms discovered in previous studies. In this review, we describe the pollen developmental processes and introduce gametophytic mutants, which form defective pollen in *Arabidopsis* and rice. In addition, we discuss the perspectives on the research on pollen longevity, adhesion and hydration.

## 1. Introduction

During the final stage of maturation, pollen grains are generally dehydrated to reach a metabolically inactive state [1]. This improves the resistance of “orthodox” pollen to environmental changes [1,2]. However, another type of pollen, known as “recalcitrant” pollen, has relatively high water content at shedding [3] and is known to be desiccation-sensitive [4]. Rice produces recalcitrant pollen grains, which become unviable within five minutes of exposure to air because of their sensitivity to desiccation [5].

In *Arabidopsis* and rice, the pollen wall is known to be a multilayered structure: (1) the intine is the innermost pollen wall and mainly comprises pectin, cellulose, hemicellulose and proteins; (2) the pollen coat fills the empty cavities of the exine and contains lipids, proteins, pigments and aromatic compounds; and (3) the exine is the outer pollen wall and contains multilayered sporopollenin [6,7]. Because the pollen wall protects pollen from different types of environments, it is an important factor that regulates hybrid seed production rate in rice [8].

**Figure 1:**
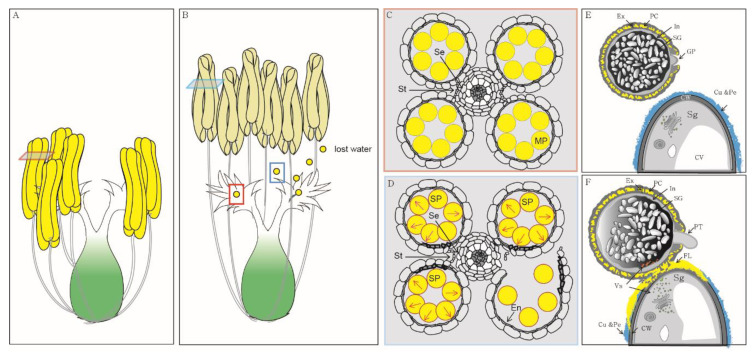
Schematic representation of a rice flower. (**A**) Flower before anthesis; (**B**) flower at anthesis; (**C**) Transverse image of an anther at the mature stage; (**D**) transverse image of an anther at anthesis. Rapid pollen swelling is the driving force behind the rupture of the anther wall. Red arrows indicate the pressure caused by swollen mature pollen grains; (**E**) pollen before landing on the stigma; (**F**) pollen after landing on the stigma. The pollen coat is mobilized on the stigma to form a “pollen foot” in rice. Numerous membranous inclusions appear on the stigma. The illustration of structural changes that occur at the point of adhesion between pollen and stigma is based on observations from *Arabidopsis*. Cu & Pe—cuticle and proteinaceous pellicle; CV—central vacuole; CW—cell wall; En—endothecium; Ex—exine; FL—foot layer; In—intine; GP—germination pore; MP—mature pollen; PC—pollen coat; PT—pollen tube; SG—starch granule; Se—septum; Sg—stigma; SP—swollen pollen; St—stomium; Vs—vesicle.

To maintain or improve the yield of rice, pollen must rapidly land on the stigma and hydrate before losing water (Figure 1A,B) [5]. The understanding of pollen behavior during pollination at cellular and molecular levels is important for improving rice yield. Because there are several recent reviews on pollen germination, in this review, we will focus on the developmental processes of rice pollen from dehiscence to hydration [9,10,11].

## 2. Pollen Swelling Is a Key Event during Anther Dehiscence in Rice

Anther dehiscence is an essential process for the release of mature pollen for pollination and fertilization. Three anther tissues, the endothecium, septum and stomium, play important roles during anther dehiscence in both *Arabidopsis* and rice (Figure 1C,D) [12,13]. Secondary wall thickening of the endothecium generates the tensile force necessary to rupture the stomium during anther wall dehydration [14,15,16]. The septum is located between the vascular bundles and two adjacent anther locules [13]. The stomium comprises a single layer of specialized epidermal cells, which has been weakened by the action of hydrolytic enzymes and is the final breakage site for anther dehiscence [13,14,17]. Several genes regulating anther dehiscence have been identified, including auxin response factor17 (*ARF17*), *MYB26*, *MYB108*, NAC secondary wall-promoting factor1 (*NST1*), NAC secondary wall-promoting factor2 (*NST2*), FT-interacting protein 7 (*OsFTIP7*), *OsYUCCA4* and homeobox1 (*OSH1*) [18,19,20,21]. Auxin negatively regulates endothecium lignification and jasmonic acid biosynthesis [22,23]. In addition, irregular xylem1 (*IRX1*), receptor-like protein kinase 2 (*RPK2*), teosinte branched1, cycloidea, PCF (TCP24), *Arabidopsis* histidine-containing phosphotransfer factor 4 (*AHP4*), secondary wall thickening-associated F-box 1 (*SAF1*), cystathionine β-synthase domain-containing protein (*CBSX2*), anther dehiscence repressor (*ADR*) and SUMO E3 ligase1 (*SIZ1*) are reported to be involved in endothecium thickening, and mutations in these genes result in non-dehiscent anthers [12,16,24,25,26,27,28,29]. *MYB21*, *MYB24* and jasmonate resistant 1 (*OsJAR1*) function in jasmonic acid-mediated anther dehiscence [30,31]. Jasmonic acid controls stomium breakage during anther dehiscence. All of these genes are involved in the biomechanical changes that occur in the anther walls to elicit successful pollen release.

In rice, the pollen itself plays important roles in anther dehiscence [32]. The rapid swelling of pollen grains drives the septum and stomium to rupture (Figure 1A,B) [32]. Increased pollen pressure results in the locule to bulge, resulting in the rupture of the septum, which has already been weakened by the action of hydrolytic enzymes (Figure 1D) [13,17]. Pollen pressure combined with the inward bending of the locule walls adjacent to the stomium results in the splitting of the stomium and the release of pollen (Figure 1D) [13,17]. Despite the importance of pollen swelling during anther dehiscence in rice, no mutant has been identified for this characteristic and its underlying mechanism remains largely unknown. Swelling by the imbibition of pollen grains is accompanied by active water movement through the water potential gradient, which is regulated by saccharides or cations [33]. Starch digestion starts three hours before flowering in rice [33,34]. This results in a decrease in osmotic potential and water uptake. In rice, anthesis is extremely sensitive to high temperatures [35]. High-temperature-induced sterility is related to defective dehiscence caused by the inhibition of pollen swelling [32,36]. In anthers with poor dehiscence that have been damaged by exposure to high temperatures at the flowering stage, decrease in both pollen volume and starch accumulation in pollen grains have been reported [32,36]. Potassium is considered as another osmoticum for rapid pollen swelling [37]. Based on the observation that potassium accumulates at the aperture of mature pollen and that it regulates water movement in guard cells, Rehman and Yun [37] hypothesized that potassium regulates rapid pollen swelling during anther dehiscence. Because pollen swelling is a specific event that occurs in several species, including rice, there is a need for research related to pollen swelling during rice anthesis to achieve successful plant reproduction.

## 3. Pollen Longevity Depends on the Structural Features of the Pollen Wall in Rice, but Not in *Arabidopsis*

After the shedding of pollen grains from anthers, pollen must land on the stigma before losing its viability. Pollen longevity is an important factor for the improvement of hybrid seed production. Up to 74% of natural outcrossing of male sterile lines has been reported, with median values of 25–35% in large-scale hybrid rice seed production plots in China [38,39]. Pollen longevity is an important factor to regulate hybrid seed production rate. While *Arabidopsis* pollen remains viable for up to three days after anthesis, rice pollen generally loses its viability within five minutes of pollen shedding [40,41]. *Arabidopsis* pollen undergoes programmed dehydration. It contains furrows that facilitate variations in the shape and volume of pollen in response to the hydration level [5].

A positive correlation between pollen longevity and pollen wall thickness has been reported in grass species [6]. The short longevity of rice pollen can be explained by its thin wall as well as by the presence of many microchannels within the exine [6]. These structural features of the rice pollen wall facilitate both fast germination on the stigma and dehydration in air, resulting in short longevity [5]. A shorter pollen longevity has been reported for the following rice mutants: *glossy1-4* (*osgl1-4*), *humidity-sensitive genetic male sterility1* (*hms1*) and *oxidosqualene cyclases 12/poaceatapetol synthase 1* (*ososc12/ospts1*) (Table 1) [42,43,44]. The defects in the pollen wall, particularly in the pollen coat, observed in these mutants result in excessive fast dehydration, leading to humidity-sensitive male sterility [42,43,44]. The pollen coat fills the cavities of the pollen exine and protects the male gametophyte from dehydration in rice. However, in *Arabidopsis,* the pollen coat has been reported to be related to pollen hydration.

The mutants of GTP-binding protein related1 (*GPR1*) and nicotinate/nicotinamide mononucleotide adenylyltransferase (*NMNAT*) exhibit shortened pollen longevity in *Arabidopsis* (Table 1) [45,46]. For example, *gpr1* pollen exhibits a thin exine and precocious germination [46]. Mature *Arabidopsis* pollen accumulates NAD+, which declines sharply after imbibition [45]. However, *nmnat* pollen cannot accumulate NAD+ and exhibits precocious pollen germination [45]. NAD+ participates in the determination of the timing of germination onset and is involved in metabolic state transition in *Arabidopsis* pollen [45]. Because *Arabidopsis* pollen is tolerant to desiccation, pollen with shortened longevity is not tightly connected with wall structure but is related to factors determining the timing of germination onset [45,46].

**Table 1:** Summary of the functionally characterized pollen associated-genes mentioned in this review.

Stage	Species	Gene	Function	Reference
Pollen longevity	rice	*OsGL1-4*	Involved in very-long-chain alkane biosynthesis in the pollen coat	[44]
		*HMS1*	Biosynthesis of very-long-chain fatty acids in the pollen coat and exine	[42]
		*OsOSC12*	Control the accumulation of fatty acids in the pollen coat	[43]
	*Arabidopsis*	*GPR1*	GTP-binding protein	[46]
		*NMNAT*	NAD biosynthesis	[45]
Pollen adhesion	*Arabidopsis*	LAP1	Produces temporary callose walls between developing microspores	[47]
		LAP3	Contains a repetitive motif found in beta-propeller enzymes	[48]
		LAP4	Cytochrome P450 CYP703A2-involved sporopollenin synthesis	[49]
		LAP5	Chalcone synthase essential for pollen exine development	[50]
		LAP6	Chalcone synthase essential for pollen exine development	[50]
Pollen hydration	rice	MLO12	MLO protein interacting with calmodulin in the cytosol	[51]
	*Arabidopsis*	GRP17	Oleosin-domain protein of the pollen coat	[52]
		EXL4	Extracellular lipase in the pollen coat	[53]
		PCP-Bs	Pollen coat protein	[54]
		CER1	Mutation causes pollen coat defect	[55]
		CER3	Mutation causes pollen coat defect	[55]
		CER6	Mutation causes pollen coat defect	[55]
		KINβγ	Regulates reactive oxygen species (ROS) levels	[56]
		SPIK	Transports potassium into the pollen	[57]
		PME48	Demethylesterification of homogalacturonan within the intine wall	[58]

## 4. Pollen Adhesion Is Mediated by Interactions between the Pollen Wall and Stigma

Pollen adheres to and hydrates on the stigma. Based on the presence or absence of a secretory fluid at the time of pollination, the stigma is classified into wet or dry stigma [59]. An interesting correlation between stigma type and pollen type has been reported by Heslop-Harrison and Shivanna [59]. Bicellular pollen is found on both wet and dry stigmas, whereas tricellular pollen is confined to dry stigmas [59,60]. Because water immediately surrounds the pollen that lands on wet stigma, the surface of wet stigma can promote the adhesion of most pollens [61]. However, the surface of dry stigma is covered with a discontinuous cuticle and proteinaceous pellicle (Figure 1E) [59].

In *Arabidopsis*, exine-defective mutants were identified via mutant screening to identify less-adherent pollen on the stigma. The results suggest that the components of the pollen exine are involved in the initial adhesion step [47,62,63,64]. Mutations in *LAP1* (callose synthase), *LAP4* (cytochrome P450 CYP703A2), *LAP5* (chalcone synthase) and *LAP6* (chalcone synthase) result in defects in both the exine structure and the adhesive strength of pollen in *Arabidopsis* (Table 1) [48,49,50,62,63,64].

After the initial exine-mediated adhesive interaction between pollen and stigma, proteins and lipids from the pollen coat are implicated in the subsequent stronger adhesive interactions with the surface of the stigma (Figure 1F) [44,65]. At this stage, the pollen coat is mobilized onto the stigmatic papilla to form a “pollen foot” between the pollen and the stigmatic surface; subsequently, the pollen coat in this region undergoes a structural change. Numerous membranous inclusions appear on the stigma, promoting water flow from the stigma to the pollen grains in *Arabidopsis* (Figure 1F) [44,66,67]. Although structural changes at the point of adhesion between the pollen and stigma have not been examined in detail in rice, a foot layer has been observed during rice pollen adhesion [44]. Because pollen adhesion rapidly occurs in rice, progress on related research has not been forthcoming for this crop [68].

## 5. Pollen Hydration Is a Pre-Requisite for Pollen Germination

Pollen hydration is necessary for the pollen to proceed to the next step of germination. Vesicle trafficking in the stigmatic papilla is one of the early cellular events associated with pollen hydration and germination (Figure 1F) [69,70]. Water, nutrients, and other small molecules are rapidly transported into the pollen grain from the stigmatic papilla [66,69].

The pollen coat is considered as an important factor in the regulation of pollen hydration because *Arabidopsis* mutants with a defective pollen coat tend to exhibit defects in the process of pollen hydration [52,53,54,55]. The presence of oleosin-domain-containing glycine-rich protein 17 (GRP17), extracellular lipase 4 (EXL4), pollen coat protein B-class (PCP-Bs) and eceriferum (CER) indicate that pollen-coat-derived molecules are required for both pollen coat development and pollen hydration in *Arabidopsis* (Table 1) [52,53,54,55]. Defects in hydration observed in these mutants are derived from a failure in the interaction between pollen and the stigma [52,53,54,55]. Pollen coat mutants block pollen hydration via defects in interactions with the stigma in *Arabidopsis*, whereas pollen coat mutants in rice show defects in pollen longevity. For example, the function of rice OsGL1-4 is likely similar to that of *Arabidopsis* CER1 [44]. A defective pollen coat was detected in both *osgl1-4* and *cer1* [44,55]. Because *Arabidopsis* pollen is of the orthodox type and rice pollen is of the recalcitrant type, different physiological effects are seen in the mutants; defective hydration is exhibited by *cer1* and short longevity is observed in *osgl1-4* towing to dehydration on the stigma [44,55].

The *Arabidopsis* KINβγ subunit of the SnRK1 complex regulates the expression of the Shaker Pollen Inward K+ channel (*SPIK*), which plays important roles in pollen hydration by regulating reactive oxygen species levels and transporting potassium into the pollen (Table 1) [10,56,57]. Pectin methylesterase 48 (AtPME48) functions to change the mechanical properties of the intine wall during maturation; therefore, mutants display delayed pollen hydration and germination [58] (Table 1).

Mildew resistance locus O12 (MLO12) from rice regulates pollen hydration, possibly via an interaction with calmodulin in the cytosol (Table 1) [51]. Although only one rice gene has been identified as being involved in pollen hydration of rice, pollen hydration on dry stigma is strictly controlled, and multiple genes may be involved in this process.

## 6. Accumulated “Omics” Data are Useful Sources of the Candidate Genes for Pollen Hydration and Germination in *Arabidopsis* and Rice

To understand pollen behavior and the underlying mechanisms that occur during pollination, several “omics” studies involving the transcriptome and proteome have been performed using the pollen in *Arabidopsis* and rice [71,72,73,74,75,76,77,78,79,80,81,82,83,84,85] (Table 2). A large number of male-gametophyte-expressed and stage-specific transcripts were identified via transcriptome analysis of both species [71,72,73,74,75,79,80,81,82]. Stage-specific and differently cellular-localized pollen proteins were identified via proteomic analysis [76,77,78,83,84,85]. Many dynamic cellular events occur during pollen germination, including calcium oscillation, vesicle transport, cell wall biosynthesis and cytoskeletal changes [81]. The gene expression profiles of mature and germinated pollens are significantly and positively correlated in both *Arabidopsis* and rice, indicating that the RNAs required for pollen germination are present in mature pollen [71]. However, the transcription inhibitor actinomycin D has an inhibitory effect on pollen germination and pollen tube growth [81,82], and pollen tube germination largely depends on the translation of stored mRNAs. Therefore, the identification of mature stage-enriched genes during pollen development is valuable for the advancement of related research. The omics data about the potential candidate genes associated with late pollen development are shown in Table 2. By comparing the transcriptomes of sporophytes with those of male gametes over time, 627 and 773 late pollen-preferred genes were identified in rice and *Arabidopsis*, respectively [71]. Comparative analysis revealed approximately 20% functional conservancy between them [71]. Genes involved in major carbohydrate metabolism were only found in rice late pollen-preferred genes, indicating a difference in the major storage reserves. Storage reserves are mainly composed of lipid bodies in *Arabidopsis* mature pollen and as starch granules in rice mature pollen [71,73].

## 7. Perspectives on the Research on Pollen Longevity, Adhesion and Hydration

Rice is a major food crop. Therefore, it is important to maintain a stable yield of this cereal. Yields from China’s hybrid rice crops are approximately 20% higher than those of high-yielding inbred varieties. However, the short longevity of rice pollen blocks hybrid seed production results in 25–35% hybrid rice seed production [38,39]. In addition, global warming is threating rice yields and anthesis is very susceptible to high temperatures [35]. Therefore, understanding pollen behavior and its underlying mechanisms are important for stable rice production. However, related researches have largely relied on studies in *Arabidopsis*. “Omics” data using pollen have accumulated, which provide a powerful tool for the identification of the global candidate genes for late pollen development in *Arabidopsis* and rice. Based on their sequence homology and expression patterns, it is estimated that approximately 20% of genes enriched during late pollen development are functionally conserved between the two species. The difference in major nutrients between rice and *Arabidopsis* in mature pollen suggests the importance of nonconserved genes in each species for understanding the molecular mechanisms. In addition, differences in pollen longevity emphasize the need for further research on rice pollen. Recently, we identified global late pollen-enriched genes in rice, and the functional significance of several candidate genes was validated by T-DNA insertional lines, showing a 1:1 segregation ratio for wild-types and heterozygotes without homozygotes. This was further confirmed by a gene editing mutant exhibiting a male sterile phenotype [71]. Therefore, accumulated omics data associated with late pollen, genome-wide gene-indexed mutant populations, and genome editing technology facilitate functional genomics studies in related research areas. Future studies in this area will shed light on the cellular and molecular mechanisms of pollen behavior. By extension, it will be possible to maintain a stable yield of rice.

## Figures and Tables

**Table 2:** Summary of the omics data, including potential candidate genes for pollen hydration and germination in rice and *Arabidopsis*.

Species	Omics Type	Accession No. ^a^	Samples	Reference
Rice	Transcriptome (microarray)		Unicellular microspore, bicellular pollen, tricellular pollen, mature pollen and germinated pollen	[71]
	Transcriptome (microarray)	GSE29080	Unicellular microspore, bicellular pollen and tricellular pollen	[72]
	Transcriptome (microarray)	GSE27988	Unicellular microspore, bicellular pollen, tricellular pollen, mature pollen and germinated pollen	[73]
	Transcriptome (microarray)	GSE17002	Mature pollen	[74]
	Transcriptome (RNA-Seq)		Unicellular microspore, bicellular pollen and tricellular pollen	[75]
	Proteome		Mature pollen and germinated pollen	[76,77]
	Proteome		Germinated pollen	[78]
	Transcriptome (RNA-Seq)	SRP022162	Mature pollen	[79]
*Arabidopsis*	Transcriptome (microarray)	GSE17343	Pollen grains (MP), germinated pollen and pollen tubes from cut pistil explants	[80]
	Transcriptome (microarray)	GSE6696	Pollen grains (MP), hydrated pollen grains and growing pollen tubes (PT)	[81]
	Transcriptome (microarray)		Mature pollen	[82]
	Proteome		Mature pollen and germinated pollen	[83]
	Proteome		Mature pollen and pollen tube	[84]
	Proteome		Mature pollen	[85]

^a^ indicates accession number of the transcriptome data deposited in the NCBI Gene Expression Omnibus or EMBL ArrayExpress [86,87].
